# Draft Genome Sequence of *Pantoea ananatis* Strain AMG521, a Rice Plant Growth-Promoting Bacterial Endophyte Isolated from the Guadalquivir Marshes in Southern Spain

**DOI:** 10.1128/genomeA.01681-15

**Published:** 2016-02-18

**Authors:** Esaú Megías, Manuel Megías, Francisco Javier Ollero, Mariangela Hungria

**Affiliations:** a Departamento de Microbiología, Facultad de Biología, Universidad de Sevilla, Sevilla, Spain; b Embrapa Recursos Genéticos e Biotecnologia, Brasília, Federal District, Brazil; c Embrapa Soja, Soil Biotechnology, Londrina, Paraná, Brazil

## Abstract

The rice endophyte *Pantoea ananatis* AMG521 shows several plant growth-promoting properties and promotes rice yield increases. Its draft genome was estimated at 4,891,568 bp with 4,704 coding sequences (CDS). The genome encodes genes for *N*-acylhomoserine lactone (AHL) synthases, AHL hydrolases, hyperadherence (*yidQ*, *yidP*, and *yidR*)*,* fusaric acid resistance, and oxidation of lignin, highlighting its biotechnological potential.

## GENOME ANNOUNCEMENT

Rice (*Oryza sativa* L.) is the dominant staple food crop and composes the main meal of more than half of the world’s population. High microbial diversity has been associated with rice, with an emphasis on a rich community of endophytes. *Pantoea* spp., belonging to the family *Enterobacteriaceae* (1), are common endophytes of rice; the genus includes species with both plant growth-promoting capacity and plant, animal, and human pathogens (2).

*Pantoea* sp. strain AMG521 was isolated as an endophyte from rice paddies at the Guadalquivir River marshes, southern Spain. After colonizing rice roots, in a few days the strain can be reisolated from surface-sterilized shoots. Studies with nonmotile mutants have shown that plant invasion by AMG521 requires flagellar motility. AMG521 shows important properties of plant growth-promoting bacteria (PGPB), including the capacity to synthesize siderophores, cellulose, and indole acetic acid (IAA) (251 mg·mL^−1^), and the capacity to solubilize phosphate *in vitro*, in addition to antagonistic activity to the plant pathogen *Xanthomonas campestris* pv. citri. Rice inoculation with AMG521 significantly increases plant growth and crop yield, indicating a high potential for its use as a commercial inoculant.

To access the bacterial genome sequence of AMG521, total DNA was extracted using the DNeasy blood and tissue kit (Qiagen) and processed on the MiSeq platform (Illumina) at Embrapa Soja, Londrina, Brazil. Shotgun sequencing allowed a 75-fold coverage, and the genome was estimated at 4,891,568 bp, assembled in 239 contigs. Sequences were submitted to RAST (3) and genome annotation identified 4,704 coding sequences (CDS), of which 52% were classified in 523 subsystems, with the predominance of the carbohydrates (24%) and amino acids and derivatives (18%) categories. The highest similarity was found with *Pantoea ananatis* strain LMG 20103.

Genes involved in Type I, IV, V, and VI secretion systems, as well as in siderophore and IAA production were detected in the genome. Two putative *N*-acylhomoserine lactone (AHL) synthase (*luxI*-like) and two *luxR*-like genes were also found, in addition to an AHL hydrolase involved in quorum quenching. Chemotaxis and motility included 125 genes. Interestingly, AMG521 carries genes involved in hyperadherence such as *yidQ*, *yidP*, and *yidR*, which might help in plant colonization.

Contributing to the safe use of AMG521 as an inoculant, we confirmed that its genome does not carry the WtsE protein, that is, an AvrE family member required by *Pantoea stewartii* subsp. *stewartii* to cause wilt and leaf blight symptoms in maize (*Zea mays* L.) (4). However, putative genes encoding for fusaric acid resistance and laccase (EC 1.10.3.2) involved in oxidizing lignin-related compounds were present and might help in biological control. Altogether, the genome confirms the high biotechnological potential of *P. ananatis* AMG521as a PGPB.

### Nucleotide sequence accession numbers.

This whole-genome shotgun project has been deposited at DDBJ/EMBL/GenBank under the accession numbers SUBID (SUB1164880), BioProject (PRJNA301127), BioSample (SAMN04235877), and Accession (LMYG00000000). The version described in this paper is the first version, LMYG01000000.
